# Brain Epitranscriptomic Analysis Revealed Altered A-to-I RNA Editing in Septic Patients

**DOI:** 10.3389/fgene.2022.887001

**Published:** 2022-04-26

**Authors:** Jing-Qian Zhang, Jia-Qi Pan, Zhi-Yuan Wei, Chun-Yan Ren, Fu-Xia Ru, Shou-Yue Xia, Yu-Shan He, Kaisheng Lin, Jian-Huan Chen

**Affiliations:** ^1^ Laboratory of Genomic and Precision Medicine, Wuxi School of Medicine, Jiangnan University, Wuxi, China; ^2^ Joint Primate Research Center for Chronic Diseases, Wuxi School of Medicine, Jiangnan University and Institute of Zoology, Guangdong Academy of Sciences, Jiangnan University, Wuxi, China; ^3^ Jiangnan University Brain Institute, Wuxi, China; ^4^ Jieyang People’s Hospital, Jieyang, China

**Keywords:** RNA editing, sepsis-associated encephalopathy, human brain, cis-regulatory analysis, epitranscriptome

## Abstract

Recent studies suggest that RNA editing is associated with impaired brain function and neurological and psychiatric disorders. However, the role of A-to-I RNA editing during sepsis-associated encephalopathy (SAE) remains unclear. In this study, we analyzed adenosine-to-inosine (A-to-I) RNA editing in postmortem brain tissues from septic patients and controls. A total of 3024 high-confidence A-to-I RNA editing sites were identified. In sepsis, there were fewer A-to-I RNA editing genes and editing sites than in controls. Among all A-to-I RNA editing sites, 42 genes showed significantly differential RNA editing, with 23 downregulated and 19 upregulated in sepsis compared to controls. Notably, more than 50% of these genes were highly expressed in the brain and potentially related to neurological diseases. Notably, cis-regulatory analysis showed that the level of RNA editing in six differentially edited genes was significantly correlated with the gene expression, including HAUS augmin-like complex subunit 2 (*HAUS2*), protein phosphatase 3 catalytic subunit beta (*PPP3CB*), hook microtubule tethering protein 3 (*HOOK3*), CUB and Sushi multiple domains 1 (*CSMD1*), methyltransferase-like 7A (*METTL7A*), and kinesin light chain 2 (*KLC2*). Furthermore, enrichment analysis showed that fewer gene functions and KEGG pathways were enriched by edited genes in sepsis compared to controls. These results revealed alteration of A-to-I RNA editing in the human brain associated with sepsis, thus providing an important basis for understanding its role in neuropathology in SAE.

## Introduction

Sepsis is a life-threatening systemic infectious disease caused by bacteria, viruses, or other factors, with high mortality worldwide (Singer et al., 2016; Rello et al., 2017; Salomao et al., 2019). Septic patients experience damage to multiple organs and systems, including sepsis-associated brain dysfunction. Sepsis-associated brain dysfunction (SABD) is also known as sepsis-associated encephalopathy (SAE). It has been found that up to 70% of patients affected with sepsis could develop SAE, which is the most common organ dysfunction in sepsis (Czempik et al., 2020). Its clinical manifestation is diverse, ranging from mild delirium to coma (Gofton and Young, 2012).

Adenosine-to-inosine (A-to-I) RNA editing is an epigenetic process of adenosine (A) to inosine (I) conversion mediated by the adenosine deaminase acting on RNA (*ADAR*s) family (Christofi and Zaravinos, 2019; Wang et al., 2020). It is recognized as guanosine (G) in reverse transcription and translation (Nishikura, 2016). A-to-I RNA editing has an important regulatory role in inflammatory diseases and neurological diseases (Gélinas et al., 2011; Chung et al., 2018). The potential role of *ADAR* has been reported in sepsis. *ADAR* is highly expressed in the small intestine of septic mice, which inhibits inflammation and plays a protective role against sepsis (Shangxun et al., 2020), providing a new potential therapeutic target for sepsis (Chen et al., 2017). Nevertheless, the role of *ADAR*-mediated A-to-I RNA editing played in sepsis remains unelucidated, especially in SAE.

Herein the current epitranscriptomic study analyzed A-to-I RNA editing from postmortem brain (the parietal cortex) tissues from septic patients and controls at the transcriptomic level and explored editing sites associated with sepsis and their cis-regulatory effects on the gene expression, providing new insight into the molecular mechanism involving A-to-I RNA editing in SAE.

## Methods

### RNA-Seq Data

RNA sequencing raw data were obtained from NCBI’s Gene Expression Omnibus (GEO) database. The dataset contained brain tissues (parietal cortex gray matter) from 12 patients who died from sepsis and 12 controls who died from noninfectious diseases (GSE135838) (Bustamante et al., 2020). Sepsis patients and controls were balanced for age, Consortium to Establish a Registry for Alzheimer’s Disease (CERAD) score, dementia diagnosis, and length of hospital stay. Detailed information can be found in the original report.

### RNA-Seq Data Alignment

The obtained sequencing data were processed as previously described (Tao et al., 2021). In brief, quality control analysis was performed using FASTQC. Alignment of reads to the reference human genome sequence (UCSC hg38) was performed using RNA STAR (version 2.7.0e) (Dobin et al., 2013), with multiple-mapped reads and deduplication removed using SAMtools (version 1.9) (Li et al., 2009), and base quality score recalibrated using GATK (version 4.1.3) (Walker et al., 2018).

### Identification and Annotation of RNA Editing Sites

RNA single-nucleotide variation (SNV) was identified using VarScan (version 2.4.3) software (Koboldt et al., 2012) using a standard pipeline described previously (Tao et al., 2021). Annotation of SNVs was performed using the Ensembl Variant Effect Predictor (VEP) (McLaren et al., 2016). Furthermore, only A-to-G SNVs with editing levels ≥ 1% observed in at least two samples or annotated as known editing variants in the REDIportal V2.0 database (Mansi et al., 2021) were retained as high-confidence variants.

### Quantification and Differential Analysis of Gene Expression

Alignment files generated by RNA STAR were analyzed using FeatureCounts to obtain counts of RNA expression (Liao et al., 2014), and normalized gene expression levels (transcript per million, TPM) were calculated.

### Enrichment Analysis of Gene Ontology and Pathways

Enrichment analysis of differentially edited genes were performed using DAVID online prediction tools (https://david.ncifcrf.gov/tools.jsp) and Enrichr (https://maayanlab.cloud/Enrichr/) with false discovery rate (FDR) < 0.05 as the significance cutoff (Kuleshov et al., 2016).

### Statistical Analysis

The intergroup levels of RNA editing or gene expression were compared using the Kruskal–Wallis (KW) non-parametric test. Frequency data were analyzed using the Fisher’s exact test. Cis-regulatory effects on RNA editing on the expression of edited genes were analyzed using the Spearman correlation to calculate the correlation coefficients *(r)* and *p*-values. Principal component analysis (PCA) was performed and visualized using R (version 3.6.3).

## Results

### A-to-I RNA Editing in Human Brain Tissues

**FIGURE 1:**
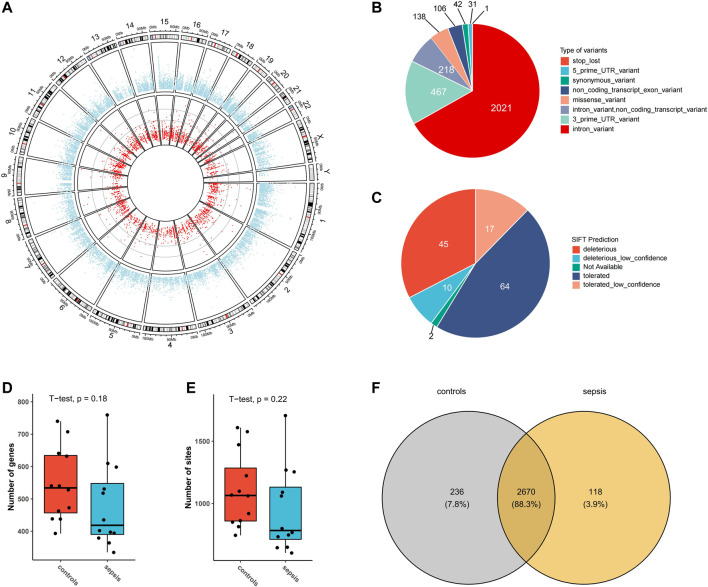
A-to-I RNA editing sites identified from human brain transcriptome in the current study. **(A)** Circos plot of transcription gene expression (outer circle) and A-to-I RNA editing sites (inner circle) in the human brain. **(B)** Functional categories of A-to-I RNA editing detected. **(C)** SIFT prediction of missense variants Boxplot of the number of editing genes **(D)** and sites **(E,F)** Venn plot of A-to-I RNA editing sites in sepsis and controls.

From transcriptomic data of the brain tissues from septic patients and controls, 3024 high-confidence A-to-I RNA editing sites in 1,192 genes were found **(** Figure 1A). These editing sites covered a variety of functional categories, including 2021 intronic variants, 467 3′-untranslated region variants (3′-UTR), 218 non-coding transcript intronic variants, 138 missense variants, 106 non-coding transcript exonic variants, 42 synonymous variants, 31 5′-untranslated region (5′-UTR) variants, and 1 stop-loss variant **(** Figure 1B). SIFT predicted 55 out of the 138 missense variants to have a potential impact on protein functions **(** Figure 1C). The expression levels of RNA editing enzymes *ADAR* and *ADARB1*, as well as the numbers of editing genes and editing sites in the brain tissues of septic patients, were lower than those in controls (Supplementary Figures S1A,B, Figures 1D,E). Of all these RNA editing sites, 118 were detected exclusively detected in septic patients and 236 in controls, and 2,670 were common in both groups (Figure 1F, Supplementary Tables S1, S2).

### Sequence Preference for Specific Editing Sites in Sepsis

The A-to-I RNA editing sites unique to the sepsis were then analyzed for sequence preference of 6 bp upstream and downstream of the editing sites. The results showed that, in most of the variant categories, G was suppressed 1 bp upstream of the editing sites. In addition, all editing sites preferred G 1 bp downstream the editing sites (Supplementary Figure S2).

### Differential A-to-I RNA Editing Between Sepsis and Controls

**FIGURE 2:**
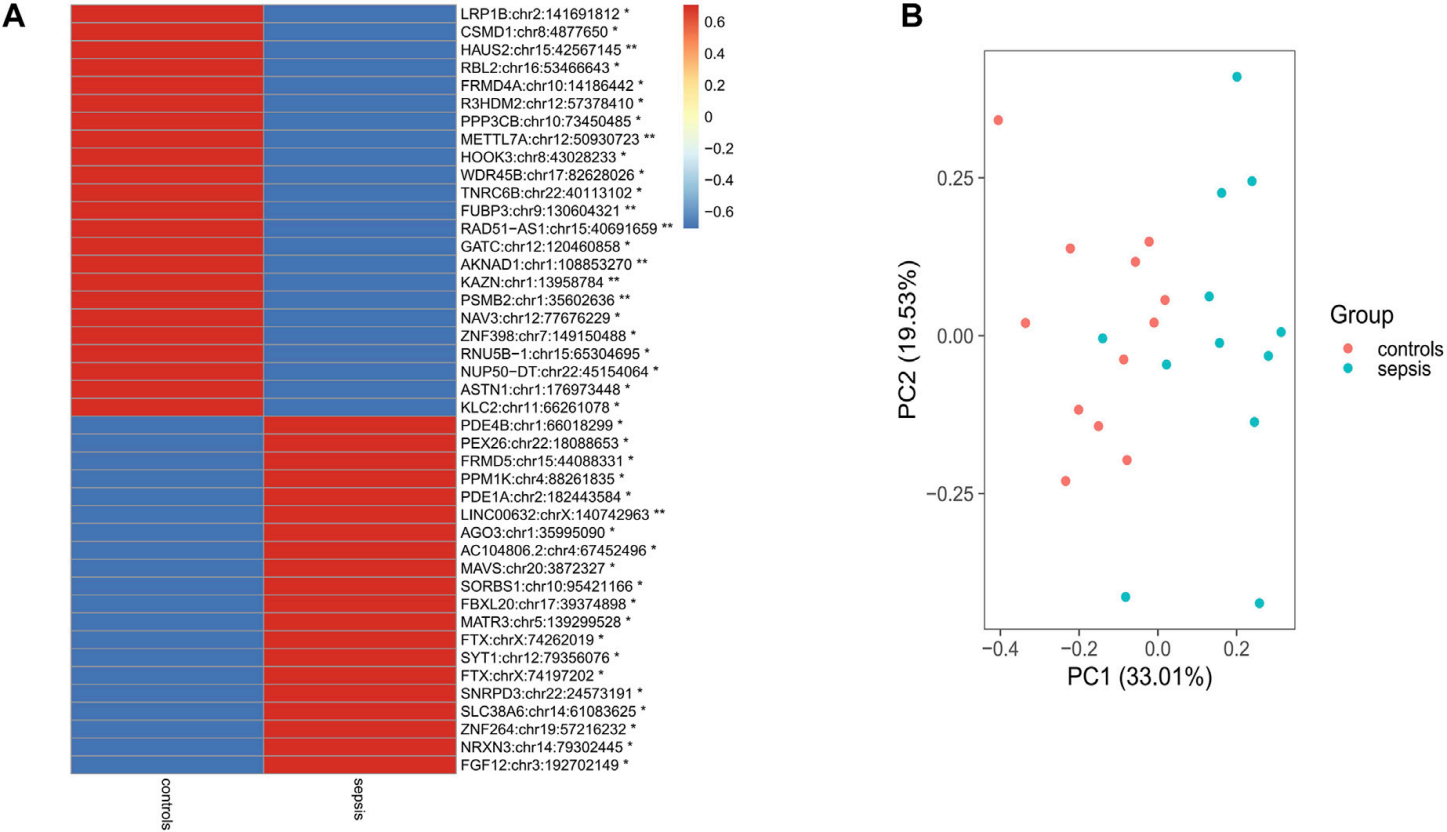
Differential RNA editing sites in the brain between sepsis and controls. **(A)** 43 sites that show statistically different editing levels. ^*:^ *p* < 0.05; ^**:^ *p* < 0.01; *p*-values are calculated using the Student’s-test. **(B)** Principal component analysis of the 43 differential editing sites between sepsis and controls.

In order to analyze differential A-to-I RNA editing in sepsis, the RNA editing levels of the sites among different groups were compared by the KW test, and a total of 43 differentially edited sites in 42 genes were found**,** with 23 genes downregulated and 19 genes upregulated in sepsis compared to controls (Figure 2A; Supplementary Tables S3, S4). Forty of these differentially edited sites were known sites. Among the 43 differentially edited sites, 23 were significantly downregulated and 20 upregulated in sepsis compared to those in controls. PCA using these differentially edited sites revealed separation of clustering between sepsis and control samples, with the contribution of PC1 and PC2 to be 33.01% and 19.53%, respectively (Figure 2B). Functional enrichment analysis of the differentially edited genes by DAVID revealed that 29 genes were related to protein binding and 16 were related to the cytosol (Supplementary Table S5). The results also showed that protein phosphatase 3 catalytic subunit beta (*PPP3CB*), kinesin light chain 2 (*KLC2*), proteasome 20S subunit beta 2 (*PSMB2*), and Matrin 3 (*MATR3*) were associated with amyotrophic lateral sclerosis, and *PPP3CB*, *KLC2*, and *PSMB2* were associated with prion disease, Alzheimer’s disease, and pathways of neurodegeneration (Supplementary Table S6), pointing to the association of sepsis with neurological damage and the important role of A-to-I RNA editing in it.

### Cis-Regulatory Effects of Differential Editing on Expression

**FIGURE 3:**
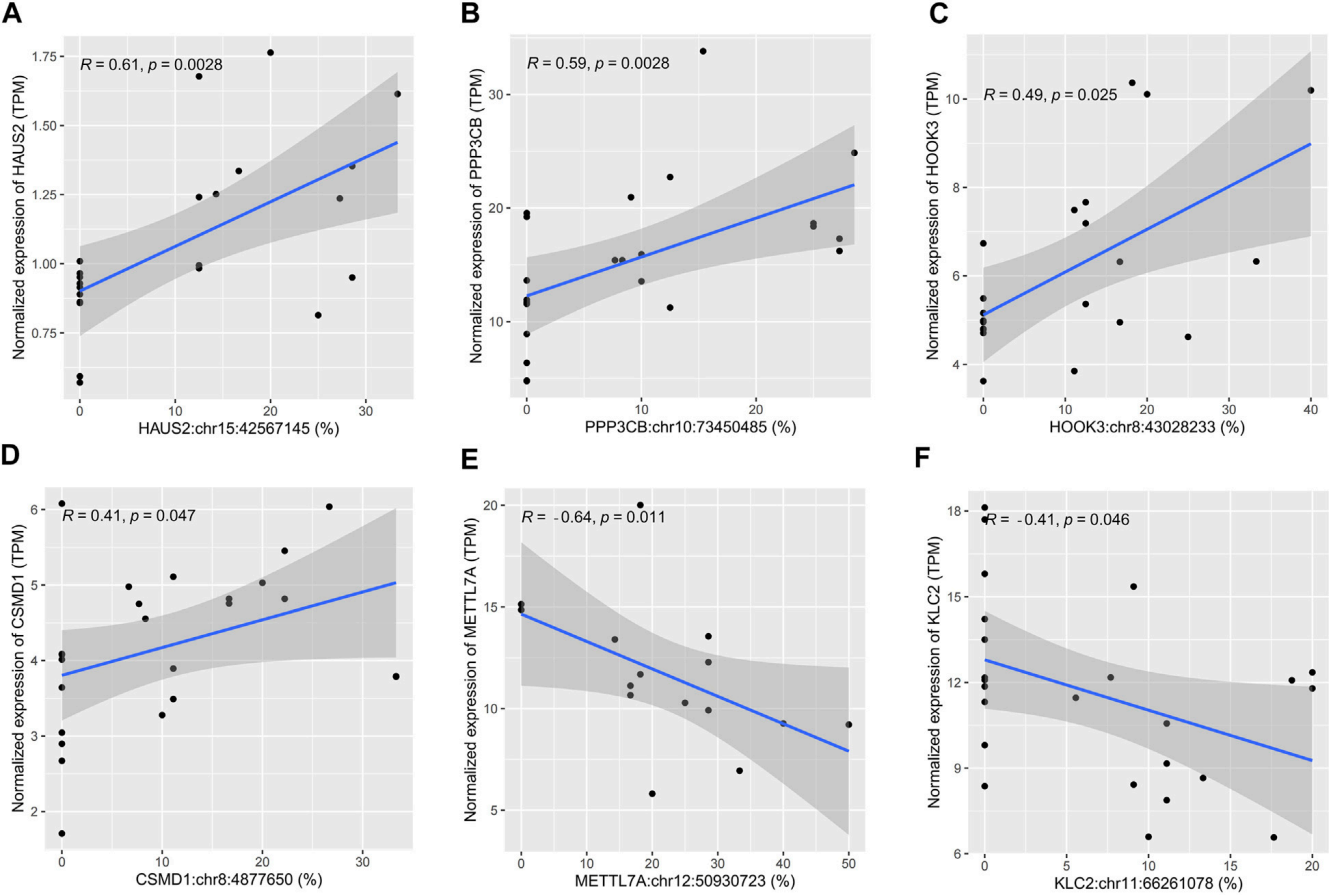
Scatter plots showing cis-regulatory effects of A-to-I RNA editing on gene expression. **(A–F)** The correlation between the editing level and gene expression level in human brain samples.

Correlation analysis between sites’ editing levels and corresponding gene expression levels was performed to investigate whether they would influence gene expression through RNA editing. A *p-*value cutoff of 0.05 was used to identify sites with higher correlation. Of the 43 differential editing sites previously found, six sites showed a correlation with the gene expression level (*p* < 0.05). Among them, the editing levels of *HAUS2*:chr15:42567145 (*r* = 0.61), *PPP3CB*:chr10:73450485 (*r* = 0.59), *HOOK3*:chr8:43028233 (*r* = 0.49), and *CSMD1*:chr8:4877650 (*r* = 0.41) were positively correlated with the gene expression level (Figures 3A–D). In contrast, *METTL7A*:chr12:50930723 (*r* = −0.64) and *KLC2*:chr11:66261078 (*r* = −0.41) were negatively correlated with the gene expression level (Figures 3E,F). In addition, these six sites all had a significantly lower editing level in sepsis than in controls: *HAUS2*:chr15:42567145 (*p* = 0.0081), *PPP3CB*:chr10:73450485 (*p* = 0.045), *HOOK3*:chr8:43028233 (*p* = 0.023), *CSMD1*:chr8:4877650 (*p* = 0.043), *METTL7A*:chr12:50930723 (*p* = 0.0049), and *KLC2*:chr11:66261078 (*p* = 0.047) (Supplementary Figures S3A–F).

### Functional Enrichment in A-to-I RNA Editing in Sepsis

**FIGURE 4:**
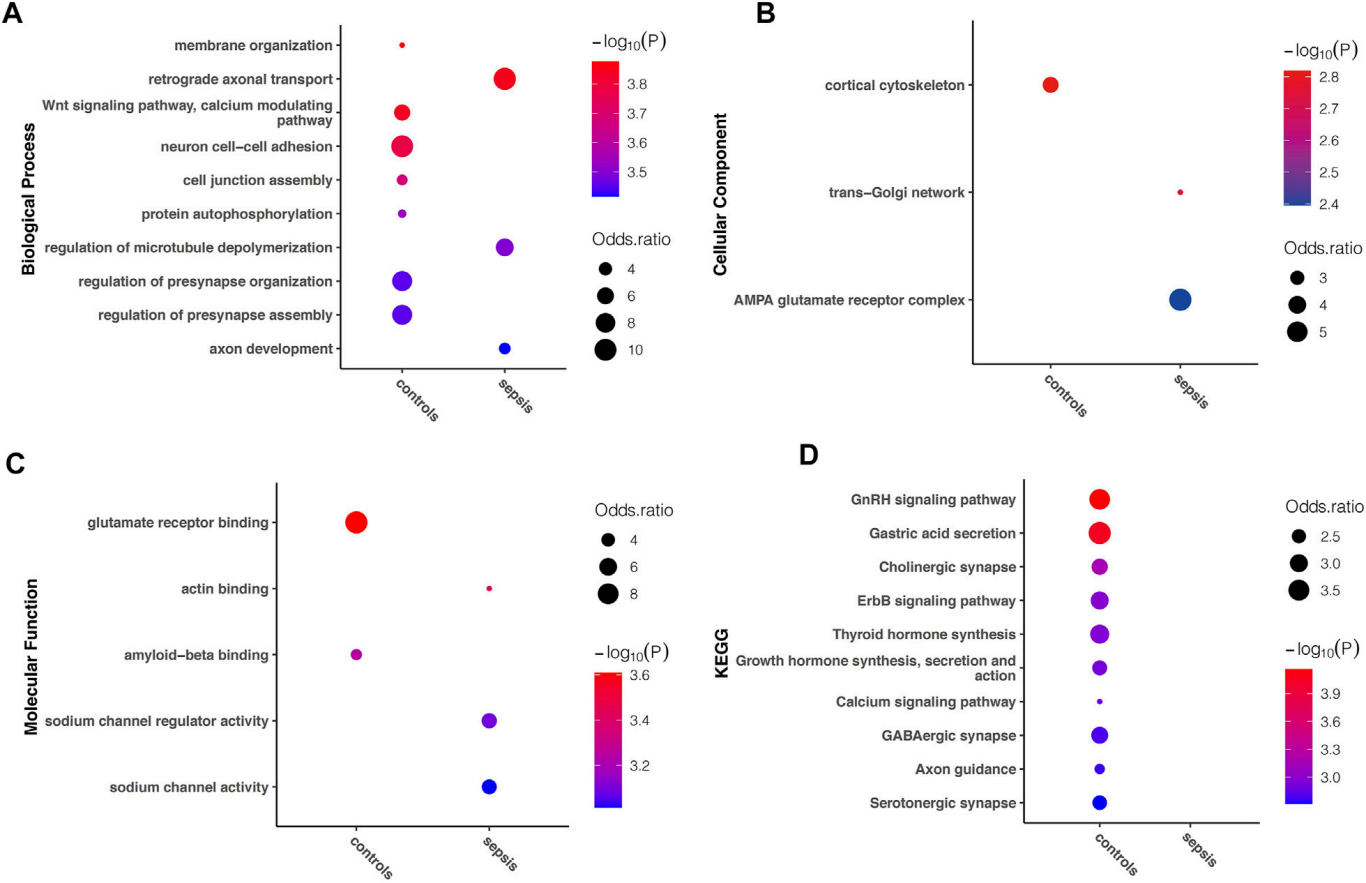
Difference in Gene Ontology and KEGG pathway enriched in sepsis and controls, respectively. The top (showing up to 10) terms with FDR < 0.05 are shown for **(A)** biological processes **(B)** molecular functions, and **(C)** cellular components, as well as **(D)** KEGG pathways uniquely enriched in either sepsis or controls.

In order to understand the biological function of A-to-I RNA editing in the human brain affected with sepsis, enrichment analysis was performed using all sites in each group. Among the top enriched GO terms, biological processes including retrograde axonal transport, regulation of microtubule depolymerization, and axon development, cellular components including trans-Golgi network, and AMPA glutamate receptor complex, and molecular functions including actin binding, sodium channel regulator activity, and sodium channel activity were unique to sepsis (Figures 4A–C). In contrast, biological processes including membrane organization, Wnt signaling pathway (calcium modulating), neuron cell–cell adhesion, cell junction assembly, protein autophosphorylation, and regulation of presynapse organization and assembly, and cellular component cortical cytoskeleton, and molecular functions including glutamate receptor binding were enriched in controls. KEGG pathway analysis revealed that numerous pathways were enriched in controls but not in sepsis, including GnRH signaling pathway, gastric acid secretion, cholinergic synapse, ErbB signaling pathway, thyroid hormone synthesis, growth hormone synthesis, secretion and action, calcium signaling pathway, GABAergic synapse, axon guidance, and serotonergic synapse (Figure 4D). Overall, a reduction of enriched gene functions and pathways in sepsis compared to controls is consistent with decreased editing enzyme expressions, and fewer editing genes and sites in sepsis.

## Discussion

Recent studies suggest that RNA editing is involved in brain dysfunction and neurological diseases. Our current study systematically investigated A-to-I RNA editing in human brain tissues and revealed its changes associated with sepsis on a transcriptome-wide scale.

It has been reported that A-to-I RNA editing is widespread in the nervous system. It is associated with the normal development of the nervous system and a variety of neurological diseases (Behm and Öhman, 2016). A-to-I RNA editing has a regulatory role in a variety of neurological diseases, such as amyotrophic lateral sclerosis, developmental epileptic encephalopathy, depression, and schizophrenia (Yang et al., 2021). In the current study, we explored the distribution of A-to-I RNA editing in sepsis-associated brain dysfunction in clinical samples. Previous studies have shown that *ADAR* is highly expressed in macrophages and has a protective effect on sepsis (Shangxun et al., 2020). Our results showed that both the levels of *ADAR* expression and A-to-I RNA editing in the brain decreased in sepsis, which could be in line with a protective role of *ADAR* and A-to-I RNA editing against sepsis.

More than 50% of the differentially edited genes in sepsis were highly expressed in the central nervous system, indicating their potential functional importance. Although no role of these RNA editing sites has been reported, the edited genes have been associated with neurological diseases. *KLC2* may exert its function through factors involved in microtubule motor activity and kinesin binding and is associated with a variety of neurological diseases such as hereditary spastic diseases, optic atrophy, and SPOAN syndrome (Hedera, 1993; Melo et al., 2015). *PPP3CB* encodes a calcium-dependent protein phosphatase that acts intracellularly on Ca (2+)-mediated signal transduction (Chen et al., 2019; Zhang et al., 2019), and its expression is significantly correlated with human brain aging (Hu et al., 2018) and glioblastoma multiforme patients’ overall survival (Lou et al., 2019). Its dysregulation has been reported in schizophrenia (Genis-Mendoza et al., 2013; He et al., 2021). Methyltransferase-like 7A (*METTL7A*) encodes a methyltransferase mainly involved in DNA methylation and the innate immune system (Lee et al., 2021). Its role in the hippocampus and neuropathic pain has been implicated (Gong et al., 2021). HAUS augmin-like complex subunit 2 (*HAUS2*) interacts with the γ-tubulin ring complex and is involved in spindle assembly (Lawo et al., 2009), and one of its paralogs are associated with glioblastoma (Ding et al., 2017). The hook microtubule tethering protein 3 (*HOOK3*) gene is involved in protein binding and microtubule binding (Kendrick et al., 2019; Wortzel et al., 2021). Its role has been implicated in neurological diseases such as Alzheimer’s disease (Herrmann et al., 2015). Expression of CUB and Sushi multiple domains 1 (*CSMD1*) is correlated with the development and treatment of schizophrenia (Liu et al., 2019). In addition, small nuclear ribonucleoprotein D3 polypeptide (*SNRPD3*) and *PSMB2* are also related to neurological diseases (Martinez and Peplow, 2017; Christodoulou et al., 2020). Notably, mutations of these differentially edited genes have been reported in neurological diseases. For example, familial ALS and distal myopathy were associated with mutations in *MATR3* (Senderek et al., 2009; Johnson et al., 2014). In addition, it has been shown that sepsis could cause long-term cognitive impairment and functional limitation in patients. *CSMD1*, *PPP3CB*, *METTL7A*, and *KLC2* have been reported to be associated with cognitive impairment or cognitive performance (Melo et al., 2015; Stepanov et al., 2017; Gong et al., 2021; Yu et al., 2021). Meanwhile, sepsis can also cause post-traumatic stress disorder and depression. *KLC2* (Du et al., 2010), *PPP3CB* (He et al., 2021), and *CSMD1* (Xu et al., 2014) were associated with mood disorders such as major depressive disorder or bipolar disorder.

Cis-regulation analysis showed that the editing level of six sites were highly correlated with the gene expression. It has been suggested that, in cancer, RNA editing can regulate mRNA abundance and thus modulate immune pathways (Chan et al., 2020). RNA editing in the 3′-UTR might affect mRNA degradation by regulating the RNA secondary structure stability or miRNA accessibility of the edited genes (Brümmer et al., 2017). One of the possible mechanisms is that the editing of *HAUS2*, *HOOK3*, and *METTL7A* mRNA may regulate their gene expression by influencing the binding of regulatory RNAs or proteins to these genes. For example, the expression of *METTL7A* as a tumor suppressor gene can be inhibited by ADAR-mediated RNA editing in the 3′-UTR (Qi et al., 2017). These results thus warranted further studies.

Gene functions and pathways of edited genes showed that the enrichment was weaker in sepsis than in controls, implicating that the sepsis-associated brain dysfunction may be related to the loss of these functions in RNA editing. Among the functions unique to sepsis, the regulation of microtubule depolymerization was noteworthy. Several studies have shown that microtubules are important in the nervous system, and their dysregulation is highly associated with neurological dysfunction (Baas and Ahmad, 2013; Diwaker and Wilson, 2019). A-to-I RNA editing could be closely related to such a biological process.

In conclusion, this study systematically investigated A-to-I RNA editing in the human brain tissues and revealed dynamic alterations in A-to-I RNA editing associated with sepsis. Our results provide a basis for further understanding how RNA editing is involved in SAE.

## Additional Information

URLs: Gene Expression Omnibus (GEO) database (https://www.ncbi.nlm.nih.gov/geo/); Ensembl Variant Effect Predictor (VEP) (https://www.ensembl.org/vep); REDIportal V2.0 database (http://srv00.recas.ba.infn.it/atlas/index.html); Enrichr (https://maayanlab.cloud/Enrichr/).

## Data Availability

The original contributions presented in the study are included in the article/Supplementary Material. further inquiries can be directed to the corresponding authors.
